# Scanning Probe Microscopy Characterization of Biomolecules enabled by Mass-Selective, Soft-landing Electrospray Ion Beam Deposition

**DOI:** 10.1002/cphc.202400419

**Published:** 2024-08-25

**Authors:** Johannes Seibel, Kelvin Anggara, Martina Delbianco, Stephan Rauschenbach

**Affiliations:** a Institute of Physical Chemistry Karlsruhe Institute of Technology Fritz-Haber Weg 2 D-76131 Karlsruhe, Germany; b Nanoscale Science Department Max Planck Institute for Solid State Research Heisenbergstr. 1 D-70569 Stuttgart, Germany; c Department of Biomolecular Systems Max Planck Institute of Colloids and Interfaces Am Mühlenberg 1 D-14476 Potsdam, Germany; d Department of Chemsitry University of Oxford OX13TA Oxford, UK

**Keywords:** Electrospray Ionization, Scanning Probe Microscopy, Glycans, Mass Spectrometry, Saccharides

## Abstract

Scanning probe microscopy (SPM), in particular at low temperature (LT) under ultra-high vacuum (UHV) conditions, offers the possibility of real-space imaging with resolution reaching the atomic level. However, its potential for the analysis of complex biological molecules has been hampered by requirements imposed by sample preparation. Transferring molecules onto surfaces in UHV is typically accomplished by thermal sublimation in vacuum. This approach however is limited by the thermal stability of the molecules, i.e. not possible for biological molecules with low vapour pressure. Bypassing this limitation, electrospray ionisation offers an alternative method to transfer molecules from solution to the gas-phase as intact molecular ions. In soft-landing electrospray ion beam deposition (ESIBD), these molecular ions are subsequently mass-selected and gently landed on surfaces which permits large and thermally fragile molecules to be analyzed by LT-UHV SPM. In this concept, we discuss how ESIBD+SPM prepares samples of complex biological molecules at a surface, offering controls of the molecular structural integrity, three-dimensional shape, and purity. These achievements unlock the analytical potential of SPM which is showcased by imaging proteins, peptides, DNA, glycans, and conjugates of these molecules, revealing details of their connectivity, conformation, and interaction that could not be accessed by any other technique.

## Introduction

In the context of molecular characterization, scanning probe microscopy (SPM), i.e. scanning tunnelling microscopy (STM) and atomic force microscopy (AFM), operated at low temperatures (LT) and under ultra-high vacuum (UHV) conditions is unprecedented in terms of real-space, spatial imaging, achieving resolution down to the sub-nanometer level,[1] in particular when using CO-modified tips.[2] In addition, scanning tunnelling spectroscopy (STS) allows for the interrogation of the local electronic properties of a molecule as well as its manipulation at the atomic scale.[3,4]

A significant barrier in the use of LT-SPM for molecular characterization is the stringent requirements of its samples: an contaminant-free, atomically flat surface on which chemically pure atomic or molecular species are adsorbed as layers, islands, or isolated particles. Transferring molecules onto surfaces in UHV for SPM imaging is often accomplished by thermal sublimation, which can be performed in-situ and hence guarantees chemical purity through the specificity of the sublimation temperature and the clean UHV environment. However, this approach is limited by the thermal stability and the vapour pressure of the molecular species, which narrows the range of molecules amenable to SPM investigations. As a result, molecules with no vapour pressure generally have been inaccessible for LT-SPM characterization due to their incompatibility with thermal sublimation-based sample preparation. Typically, sublimation is not possible for large molecules with multiple functional groups, which inhibits LT-SPM studies of most biomolecules like proteins, peptides, DNA, glycans as well as conjugates of these molecules. So far, only small thermally stable biomolecules like amino acids,[5,6] nucleobases[7–9] or porphyrins[10] have been accessible via thermal evaporation and have been studied by UHV SPM.

As an alternative to thermal sublimation, electrospray ionization (ESI) offers transfers of molecules from the solution phase to the gas phase regardless of thermal stability.[11,12] The soft ionization of ESI together with its versatility towards most types of molecules made ESI-based mass spectrometry as one of the most important tools for chemical analysis in many branches of science,[13] revealing the chemical identity and interactions of biomolecules as well as synthetic molecules.[11]

Molecular ion beams generated by ESI can not only be detected in a mass analyser, but can also be guided onto a surface in vacuum for surface deposition. This approach is known as preparative mass spectrometry, ion soft-landing,[14] or electrospray ion beam deposition (ESIBD)[15]. Typically, a mass filter is employed to select the molecular species to be directed at the surface and the collision energy is controlled to ensure intact landing and adsorption of the molecule.[14–18] Unfiltered ions from ESI sources can also be transferred into vacuum and used for deposition on a surface, in an approach distinguished as electrospray deposition (ESD).[19]

In this concept, we show that ESIBD (and also ESD) is ideally suited as preparative method for SPM. Together, ESIBD+SPM have great potential as a general analytical tool for the investigation of biological molecules. Both methods enable the deposition of complex organic molecules onto a surface under UHV conditions and their subsequent SPM investigation. With these new approaches, SPM emerge as a new tool to address the analytical challenges related to chemical composition, conformation, and interactions of biological molecules such as proteins, peptides, nucleic acids, glycans as well as conjugates of these molecules.

### Electrospray Ion Beam Deposition (ESIBD) and Electrospray Deposition (ESD)

Biological molecules generally dissolve well in aqueous solution from where they can be brought into the gas phase via electrospray ionisation (ESI) as intact, molecular, gas-phase ions.[11,12] Because ESI requires an ambient pressure environment, using these ions for surface deposition in vacuum demands an air-vacuum interface to transport them from ambient conditions to the sample in UHV. To this end two approaches exist: (i) electrospray ion beam deposition, ESIBD[14–18] (often also called ion soft landing) and (ii) electrospray deposition in high-vacuum, ESD. The key elements for the two approaches are sketched in Scheme 1.

Both methods rely on electrospray ionisation (ESI) to create gas-phase particles of the molecular analytes for surface deposition. ESI is relatively simple to operate: a high voltage (approx. 1 kV) between an emitter needle and a counter electrode, usually the vacuum inlet, will generate a spray in which ions are created from a cascade of Coulomb explosions (droplet fission) as they become unstable due to solvent evaporation while charge is retained. The choice of flow rate, emitter geometry, and solution preparation can have a profound impact on the yield and state of the ions, in particular for biological molecules.[20] Most importantly, low voltage, sharp emitters, low flow rates, and the use of volatile buffers, among other parameters, can preserve native conformations and non-covalent complexes, while organic solvents, extreme pH, and higher voltages would yield higher current but are likely to affect native conformation and complex stoichiometry.[21–23]

Following the ionisation at ambient pressure, a capillary (CAP) is used to transfer the generated ion cloud into the first vacuum chamber. Further, differential pumping is employed, i.e. several vacuum stages interconnected by small apertures to reduce the pressure by pumping the carrier gas while letting ions pass to the next chamber. Beyond the commonality of the use of ESI to create molecular ions, a vacuum inlet capillary, and differential pumping, ESIBD and ESD differ in the way the ions are transported to the sample and in the landing process. ESD relies on a stack of three aligned skimmers (SK1-3).[19] These remove gas from the supersonic jet generated when the gas leaves the transfer capillary, passing a collimated gas jet downstream in which the heavy molecules are enriched. After three skimmers the jet impacts on the sample and the particles are deposited, the impact energy defined trough the velocity of the particles and likely in the range of sound velocity.

Scheme 1. Schematics of typical ESIBD (a) and ESD (b) setups. In both cases, the ion beam is created by spraying a solution containing the ions at ambient pressure (ESI). In ESIBD, a quadrupole mass-mass filter (QUAD) is used to select the desired m/z. Ion optics (IF and LENS) are used to guide the ion beam. A mass spectrometer (MS) is used to analyse the beam composition. In ESD, a capillary (CAP) and skimmers (SK) are used to guide the ions onto the surface. Differential pumping stages are required to reduce the pressure from ambient to UHV along the ion beam.

ESD provides a compact and relatively easy to operate deposition setup, but it can result in the co-deposition of impurities from solution and solvent molecules, which may require additional heating of the sample after deposition.[24] During deposition a (static) pressure of 10-7 mbar measured in the deposition chamber and further the jet may contain, droplets, and solvent vapour in addition to the molecules, which all directly impact the surface. Therefore ESD is usually used with inert surfaces such as Au(111), followed by a gentle annealing to desorb volatile solvent molecules or impurities on surface. Beyond the examples for biological molecules given below, using ESD the deposition and imaging of macromolecules such as C60,[25,26] conjugated polymers,[27] molecular magnet Mn12 acetate,[28] and carbon nanotubes[29] have been demonstrated.

ESIBD, in contrast, relies on charged particle optics to transmit ions controlled through the differential pumping stages to the sample. As the pressure decreases, different ion optics have to be used. At high pressures (10-1 mbar), radio-frequency (rf) ion funnels (IF) and ion guides are used, whereas, at low pressures (below 10-3 mbar) electrostatic ion optics (LENS) are used to focus and steer the beam, but also rf ion guides can be employed. Usually, a quadrupole mass-filter (QUAD), also in high vacuum, provides the chemical selectivity for the method, sometimes in conjunction with an additional high-resolution mass analyser (MS) to monitor the chemical composition after filtering.[15,30] The well-defined, thermalized ion beam in ESIBD allows for the use of electrometers to monitor the molecular ion current, typically at the sample holders and apertures, which is used to measure coverage.

ESIBD deposition occurs at a sample holder to which a voltage is applied to adjust the incident energy in the range from 0.5 eV up to several 100 eV per charge. To set the desired landing energy, retarding-grid energy analysers are first used in ESIBD to measure the kinetic energy distribution of the ions. For soft-landing conditions, i.e. landing of the intact molecules without fragmentation, the ions are landed with an energy below 5 eV per charge. Higher energies can be used to specifically induce fragmentation upon surface collision[31] or reactions with the surface.[32]

Generally, the vacuum system of an ESIBD source is more complex as compared to ESD, for instance containing one or two more differential pumping stages to enable deposition in UHV (10-10 mbar). At this pressure the sample can be held at low temperatures during deposition without risking major contamination due to the adsorption of residual gases on the surface. This allows to use also more reactive surfaces like Cu or even deposition on catalytically active surfaces like TiO2-anatase has been demonstrated.[33]

One drawback of ESIBD is the intense continuous pumping required for the differential vacuum, whose noise could be prohibitive to SPM measurements. Because of this, the SPM system is typically vibrationally isolated from the ESIBD system, where samples could be transferred after deposition under vacuum using a UHV-suitcase.[34,35]

It should be noted that the charge-state of the deposited molecules can affect the adsorption behavior. For a negatively charged deprotonated ion, such as carboxylate (RCOO-1) or alkoxide (RCO-1), the formation of a covalent bond between the deprotonated site and surface is expected. As an example, in the landing of deprotonated oligosaccharides on Cu-surface, the alkoxide group promptly formed a O-Cu covalent bond upon contacting the surface. For a positively charged, protonated ion, such as amine (NH3+), the final state of the ion on surface should strongly depend on the alignment of LUMO with respect to the Fermi level of the metal. If the LUMO of the protonated molecule is lower than the Fermi level of the metal, a surface-to-molecule charge transfer could occur to deprotonate the NH3+ moiety to adsorbed NH2 and adsorbed H-atom. Alternatively, if the LUMO of the protonated molecule is higher than the Fermi level of the metal, the protonated moiety should remain in the molecule, where the protonated moiety is stabilized by a corresponding negative image charge on the metal surface.

### Imaging the primary structure of individual biopolymers

**Figure 1:**
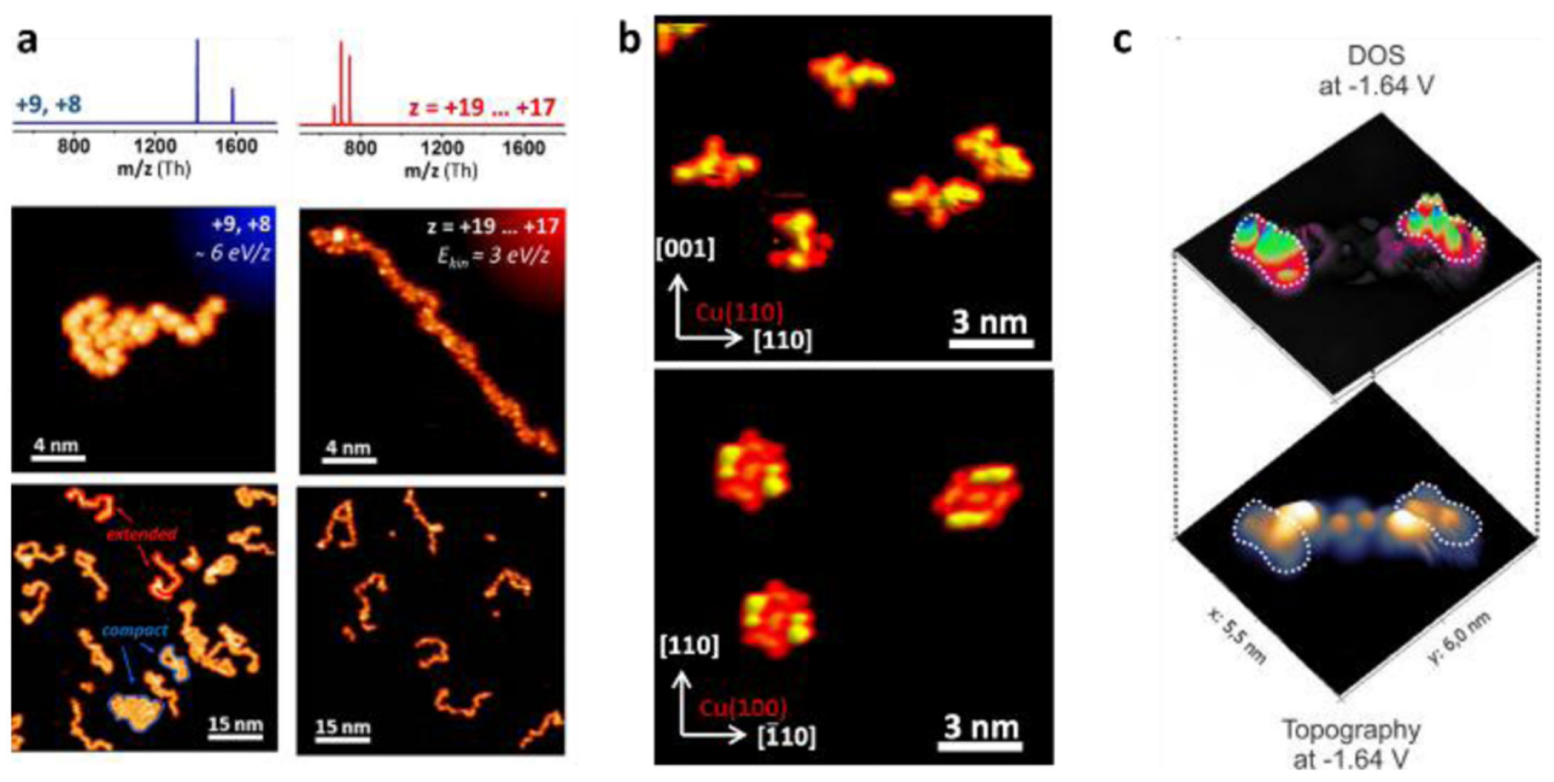
STM imaging of peptides. (a) STM images of unfolded cytochrome C deposited in different charge states Conformation control is achieved by selecting the charge state, i.e. high charge states result in elongated conformation (left), low charge states lead to compact conformations (right). [37] (b) Conformational variability of Bradykinin on Cu(110) and its regular 2D folding on Cu(100).[38] (c) A molecular modified STM tip yields chemical information about one amino acid using tunnelling spectroscopy imaging.[39] Adapted with permission from ref. [37]. Copyright (2014) American Chemical Socienty (a). Adapted with permission from ref. [38]. Copyright (2017) American Chemical Socienty (b).

**Figure 2:**
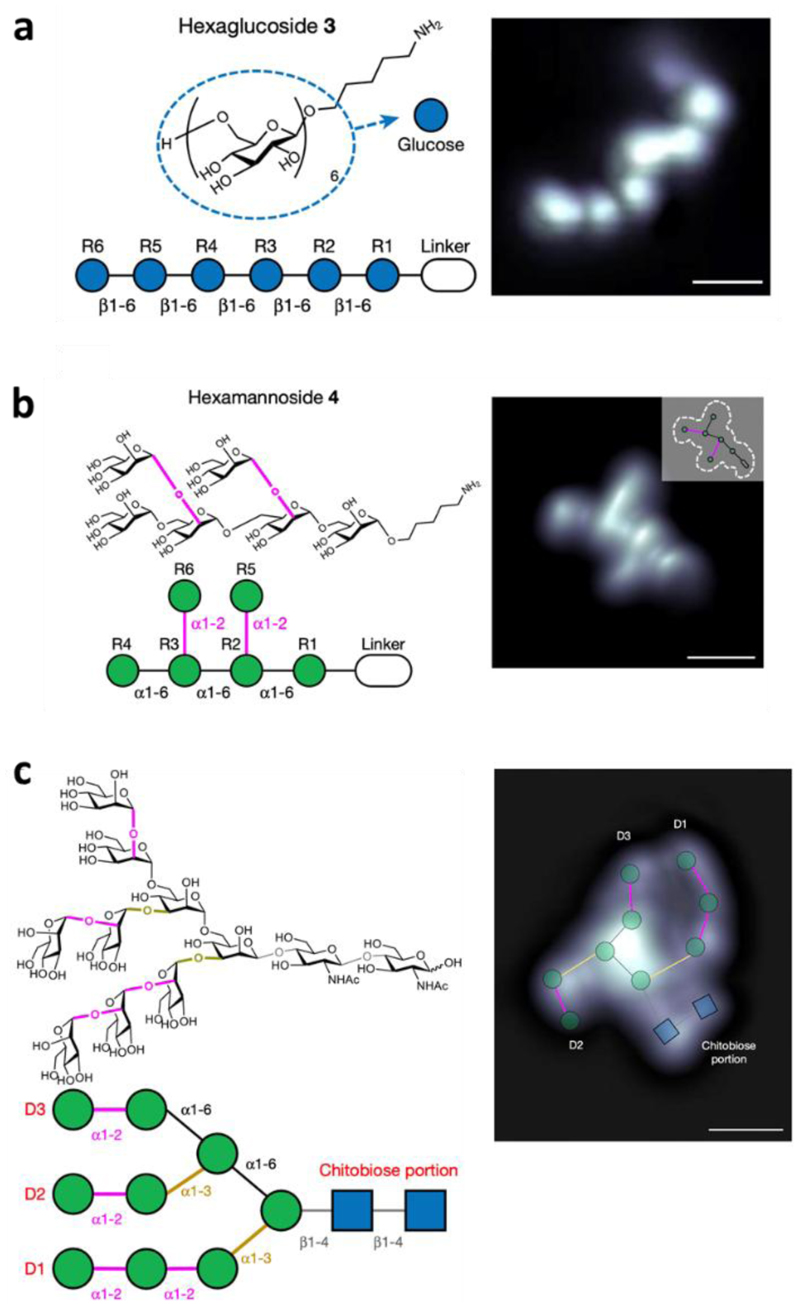
STM imaging of individual glycans. The STM images show single glycan molecules deposited onto Cu(100) held at 120 K. Single monosaccharide units can be identified in linear molecules of different lengths (a). In branched molecules, the branching points can be identified in addition to the monosaccharide units (b and c).[34]

Imaging of individual molecules on surface has been accomplished by immobilizing them upon landing on a less inert surface, such as Cu(100), or by deposition at low temperature. Examples are shown for proteins shown in Figure 1 and glycans in Figure 2. Generally, the molecules are imaged with a high contrast from the underlying surface, where the molecules are 100-200 pm in height when unfolded, or 1-3 nm in height when folded, and at a lateral resolution of below 1 nm, where molecular subunits and their shapes can be resolved. This resolution is sufficient to recognize individual amino acids or monosaccharides, whereas more spatial details are only revealed by imaging with non-contact AFM or STM using CO modified probes.

Figure 1a shows STM images of individual unfolded proteins (cytochrome c) deposited by ESIBD under different conditions onto different surfaces.[36,37] While they present a variety of conformations (their origin will be discussed in the following section in detail), for most of them the characteristic shape of the polymer strand is clearly distinguishable, with the exception of the two- and three-dimensionally folded species (not shown). Along the peptide strand a number of protrusions is seen, which can be identified as the sequence of amino acids, appearing different in size and shape. However, the sequence’s chemical information cannot be inferred directly from the STM image, because the appearance of each feature depends on the identity of the amino acid, but also on the conformation of the monomer that could be influenced by interactions with neighbouring groups. This is illustrated by the STM images of individually adsorbed Bradykinin[38] where the location of the two phenylalanine units can be determined in some configurations based on the extended shape of the sidechain and the low height of the phenyl group (Fig. 1b).

Therefore, obtaining chemical information needs to rely on alternative approaches. For instance, recently it was demonstrated that tunnelling spectroscopy with molecular modified probes has the capability to enhance chemical contrast along a polypeptide chain (Fig. 1c).[39] Furthermore, machine learning algorithms may be trained to identify sequence information from the complex patterns of features, as recently demonstrated for synthetic molecules.[40]

A prime example to showcase the analytical potential of ESIBD are the recent STM studies of glycans[34] and glycoconjugates.[41] Glycans are made from over 100 different monosaccharides, which can be connected with multiple regio- and stereochemistry, and further modified with functional groups.[42] These combinations forms a huge portfolio of linear as well as branched structures. Moreover, glycans are often connected to other biomolecules (e.g. proteins), forming glycoconjugates which, in turn, appear in different glycoforms, i.e. biomolecules with different glycans attached. Due to the vast complexity of glycan and glycoconjugate sequences, paired with the chemical similarity of the monosaccharide building blocks, the identification and sequencing remains a major analytical challenge.[43,44] Another hurdle lies in determining the secondary structure, i.e. glycan conformation.[45,46] Glycans are flexible molecules that can adopt multiple conformations, which are separated by small energy barriers, and conventional analytical techniques only provide an ensemble average of all these conformers.

Soft-landing ESIBD in combination with low temperature UHV SPM recently successfully tackled these analytical challenges. Low temperature during deposition and measurement immobilises and isolates the molecules on the surface, while effectively freezing molecular flexibility. Single molecule imaging by SPM then visualizes individual glycan[34] and glycoconjugate structures.[41] Insights into the primary sequence and branching were gained from images resolved at the single molecule level. By observing the conformation of many glycan molecules, information on the configurational variability, energy landscape, mechanical properties of glycans were gained.[47,48] Lastly, the fabrication and imaging of molecular assemblies of glycans yielded information on intermolecular interactions, e.g. on the position intermolecular hydrogen bonds.[49]

**Figure 3:**
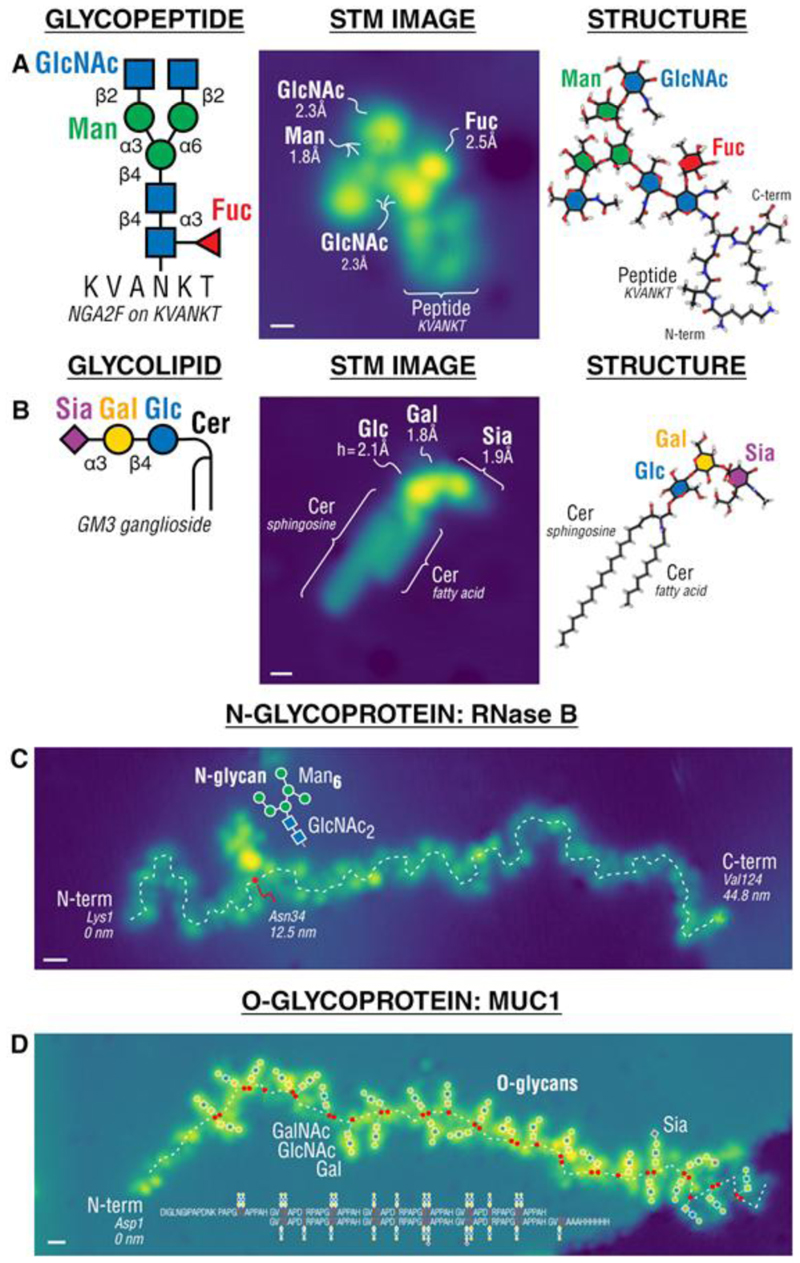
Glycoconjugates. STM images of single glycopeptides (a) and glycolipids (b) and glycoproteins, allowing the differentiation between the glycan and peptide or lipid part of the molecule. STM images of glycoproteins (c and d) reveal the number, structure and attachment site of glycans bound to the protein.[41]

Expanding on this approach of ESIBD+STM imaging has recently been shown to give unprecedent structural details of glycoconjugates at the single-molecule level.[41] The characteristic difference between peptide and glycan part in a glycopeptide is apparent due to the slightly larger height and size of the glycan (Figure 3a). For glycolipids, the characteristic appearance of the aliphatic chain of the fatty acid residues is intuitively apparent (Figure 3b). Moreover, even subtle modifications such as sulfate groups can be detected (Figure 3c). The identification and assignment are guided by the precise chemical information from mass spectrometry and mass selection and can be confirmed by DFT simulations.

STM imaging with sub-molecular resolution allowed the identification of the structure and attachment site of the glycans on a glycosylated protein (Figure 3d). For this the proteins were deposited from high charge states to ensure an extended conformation to reveal the entire sequence. Then even the multiple glycosylated proteoforms can be imaged and the glycosylation sites can be assigned (Figure 3e).

So far, studies on carbohydrates were predominantly performed with STM, while high-resolution AFM imaging is still lacking, which has the potential to give access to additional structural details. Force spectroscopy by lifting glycan chains from the surface could give information on binding energies of monosaccharide units, similar to force spectroscopy performed with ssDNA.[24] Biomolecular conformations on surfaces in vacuum

Figures 2 and 3 show proteins adsorbed on surfaces in a variety of conformations. The adsorption conformation of a protein, or more generally any biopolymer, is determined by gas-phase conformation, landing process, and surface interaction. For peptides of only few amino acids (AA), regular structures are observed after a deposition, whereas longer polypeptide chains generally show random polymer configurations.[36,38] In ESIBD conformations can be actively controlled through experimental parameters of the deposition, as summarised in Table 1 below.

In ESIBD and partly also in ESD, these parameters can be acted upon and in a variety of experiments. The control over biomolecular conformation has been demonstrated and imaged in STM. For cytochrome C deposited via ESIBD on weakly interacting substrates like Au(111) it has been shown that the protein folds randomly in two dimensions forming compact patches, while extended chain-like structures can be observed on the more strongly interacting Cu(100) where the gas-phase conformation converted into a 2D conformation and immediately immobilised.[36] In the same setting, it was shown that the charge state of the deposited protein affects the conformation.[37] Protein ion beams contain many charge states, for cytochrome C ranging from +8 to +19. The selection of a high or low m/z-range led to extended or compact conformations, respectively, which is consistent with increasing Coulomb repulsion leading to stiff molecules that do not buckle upon deposition. Additionally, the landing energy affected the final on-surface conformation, where a higher landing energy resulted in more compact structures observed for the molecules of high charge states.

Understanding how the dynamics of the landing process shape the outcome, ESIBD can be used to study the origin of molecular conformation.[20] Studies on single, chemically modified carbohydrate molecules landed on Cu(100) at 120 K revealed the local flexibility caused by severing hydrogen bonds between the monosaccharide units.[48] Soft-landing with very low energies between 5 eV down 0.5 eV enabled the exploration of the molecular conformation space of a hexasaccharide.[47] That is, cellohexaose, a hexasaccharide consisting of six β-1,4 linked glucose monomers, adopts a coiled structure in the gas phase and remains predominantly coiled when landing with an energy of 0.5 eV, but extends to more linear structures when landing at higher energies. (see Fig. 2). This method opens up new avenues for the characterization of glycan conformations, another hurdle of these biomolecules.[45,46] Here we show that ESIBD can be used to select different conformations and explore the different landscape of conformers that a glycan can adopt.

**Figure 4:**
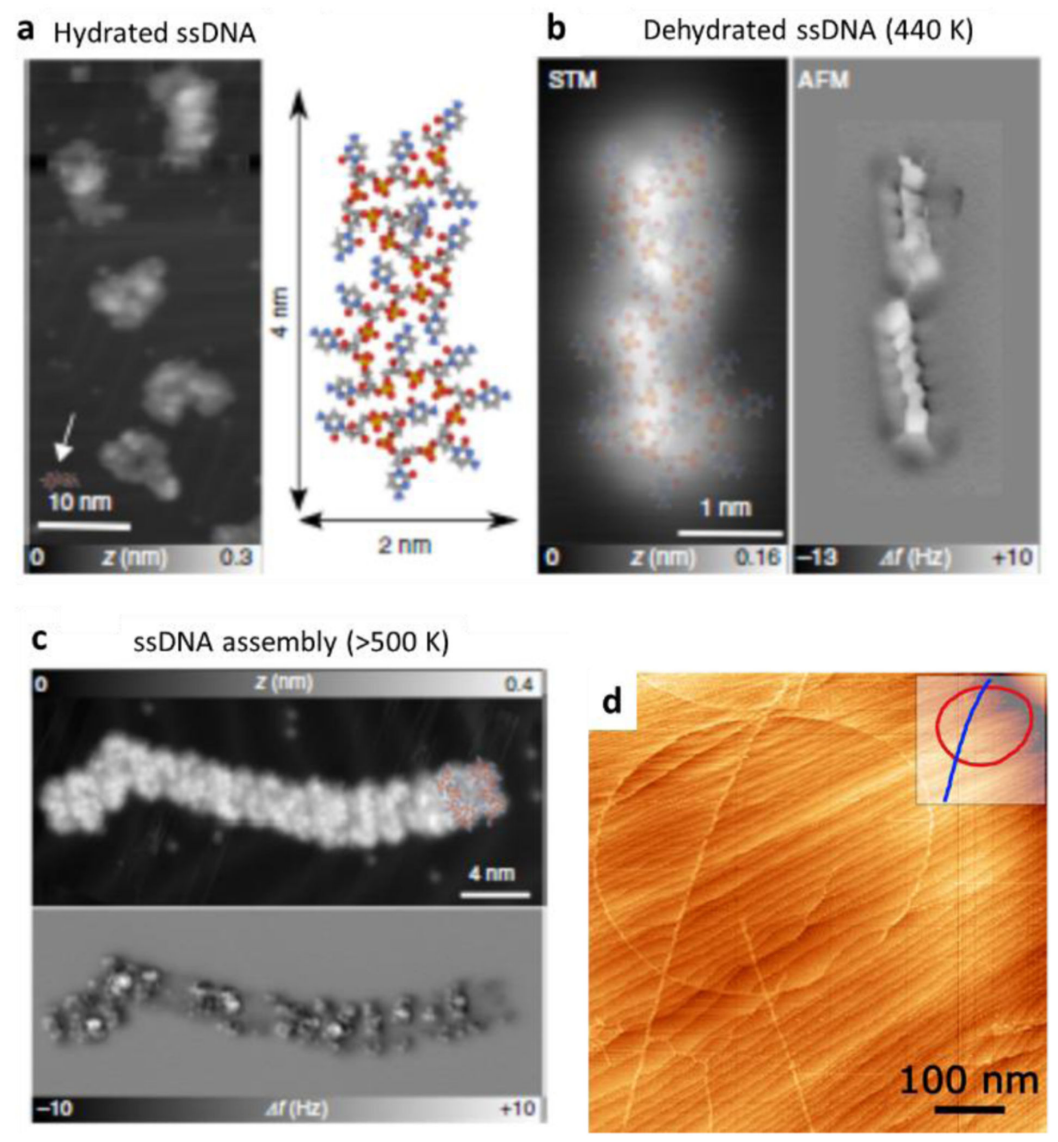
ESIBD and ESD of DNA. AFM and STM images showing short ssDNA on Au(111) prepared by ESD at room temperature (a) and annealed to 440 K (b) and above 500 K (c). Hydrated ssDNA adopts a coiled structure (a), but elongates after dehydration (b). Annealing above 500 K results in the formation of self-assembled linear structures of parallel linear ssDNA (c). [24] Large plasmid DNA (1.7 MDa mass) has been shown to remain intact after ESIBD on Ag(111).[17]

Large plasmid DNA with a mass of 1.7 MDa has been deposited using ESIBD on Ag(111) (Fig. 4c). At low coverage, STM imaging showed the intact circular plasmid structure as well as supercoiled rod-like structures.[17] ESD has been employed to deposit single strand DNA on Au(111). Subsequent high-resolution atomic force microscopy (AFM) revealed different folding conformations depending on the annealing temperature of the sample (Figure 4a-c). Force spectroscopy showed dips every 0.2-0.3 nm, which was attributed to the sequential detachment of single nucleotides during lifting.[24] These studies of DNA taken together reveal how single stranded DNA adopts new conformations on the surface in vacuum readily, while double stranded DNA is highly stable.

### Intermolecular Interactions and Assembly

Because interactions between biological molecules drive biology it is important to unveil the structural basis of these interactions. Ideally, ESIBD combines chemical information from mass spectrometry with detailed structural data from microscopy into a clear picture of an interaction. Together with SPM imaging, ESIBD+SPM could provide a preliminary idea on how molecules interact, which could serve as a model system to understand interactions in biological systems.

SPM is best when imaging quasi two-dimensional molecules and their assemblies, revealing interactions at the level of the single proton.[50,51] However, due to the absence of water, the conformation of biological molecules on surfaces in vacuum is unlikely the same as in native environments, Also, native biological molecules are distinctly three-dimensional and therefore structural investigation by electron microscopy methods, such as cryoEM[52] or low energy electron holography (LEEH),[53] are more appropriate to gain information on the 3D structures of protein samples prepared by ESIBD.

Still, fundamental aspects of molecular assembly, such as interaction motives, influence of mobility and flexibility, distribution of polar and non-polar groups, bond strength hierarchy, or steric availability of binding sites can be studied at great detail. To this end the molecular assembly of peptides and glycans has been studied using ESIBD+SPM.

**Figure 5:**
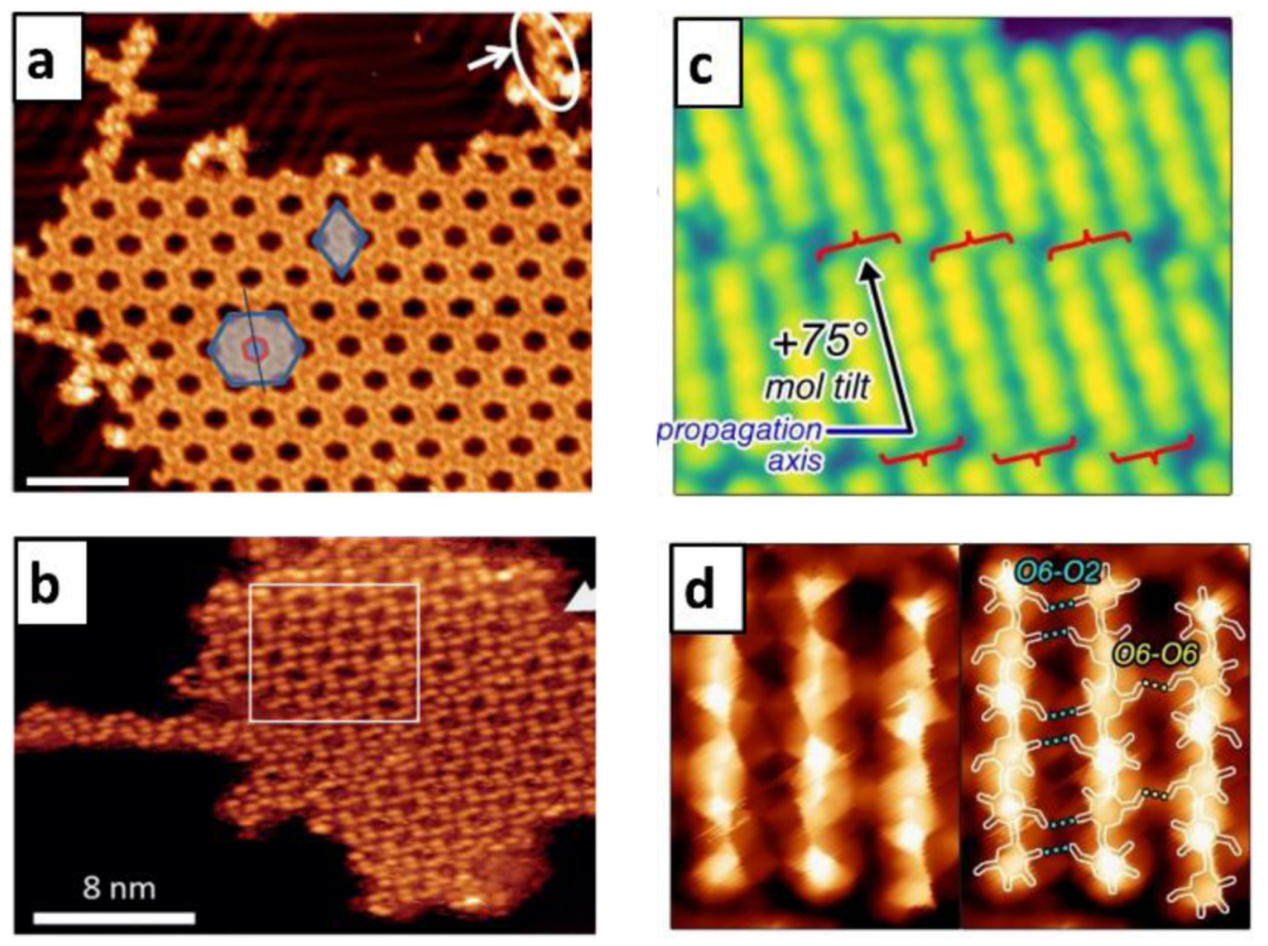
Imaging of interactions of biological molecules. STM images showing network formed on Au(111) by the peptide angiotensin II (a).[54] The network shown in b is formed by sucrose deposited onto Cu(100).[55] STM images of cellohexaose on Au(111) reveal dimer formation (c). Using CO-modified tips, the resolution of the imaging could be significantly improved, revealing additional details in the contrast between the molecules, which indicates the positions of intermolecular hydrogen bonds (d).[49]

While proteins at surfaces form random structures, short peptides are found to form ordered structures. Depending on the interaction with the substrate, long-range ordered crystalline 2D-patterns were found as well as agglomerations in 1D and peptide dimers (Fig. 1b and 5a).[54] These structures have in common that the peptide tends to arrange such that the polar groups are at the inside of the structure, while the unpolar groups are presented at the rim, opposite to the behaviour in water. Hence the absence of water in the self-assembly shifts the strength of the interactions towards the polar bonds due to missing hydrophobic effect and a lack of polar binding partners in the solvent (Fig. 1b).[56] Similarly, the molecular assembly of glycans has been studied. The disaccharides sucrose[55] and trehalose[57] on Cu(100) showed self-assembly of periodic structures. (Fig. 5b).

With the significant improvement in resolution brought by using CO-functionalized STM tips, the binding motifs of cellohexaose were observed in great detail.[58] Revealing these binding motifs is immediately relevant for the understanding of materials based on cellulose, one of the most abundant plant-based materials used in technology.

Cellohexaose molecules were deposited on Au(111) held at room temperature for increased molecular mobility, resulting in the formation of ordered structures consisting of dimers (Fig. 5c).[49] The high-resolution CO-tip imaging revealed details in the intramolecular arrangement (Figure 6b,c). Glycosidic bonds showed different contrasts depending on the orientation of the oxygen. Further, intermolecular contrast revealed the position of hydrogen bonds between neighbouring molecules (Fig. 5d). Additionally, the co-deposition of D– and L– cellohexaose showed separation of carbohydrate enantiomers in the crystallization on the surface.

### Summary and Outlook

The recent work presented here has shown the potential of combining soft-landing ESIBD or ESD and for biomolecule characterization by SPM. Just like ESI has opened possibilities for mass spectrometry, the application of SPM to high resolution imaging can now be combined with tangible application questions that would be difficult to tackle with other analytical means. The real-space imaging capabilities of SPM on the single-molecule level have been proven to be particularly relevant for carbohydrate and glycoprotein characterization, where ensemble averaging methods are severely limited by molecular complexity and flexibility. Other biomolecules of interest with similar limitations for today’s common analytical methods include glycoRNAs, lipopolysaccharides, and proteoglycans.

The deposition on insulating layers like NaCl, MgO or h-BN may help to further increase the resolution of STM and AFM imaging. Ultimately, high-resolution imaging with functionalized tips may be used to determine the amino acid sequence in proteins, monosaccharide units in carbohydrates and even identify proteins and carbohydrates in unknown mixtures.

Even though SPM is limited in interrogating 3D structures, for the study of molecular assembly and intermolecular interactions a host of new synthetic and natural molecules are now available due to the capabilities of ESI. In particular, the work is not limited by thermal stability any more, which means that now molecules of large size, complex functionality, and even highly reactive species can be used in assembly experiments.

To bridge the gap to the native environment, self-assembly could be studies in the presence of solvent molecules, to mimic native environments, which offers the chance to study solvation at the molecular level.[52,59]

Beyond the characterization of single-molecules, co-deposition of different species, e.g. peptides and carbohydrates deposited either parallel or subsequently, in combination with high-resolution imaging can reveal intermolecular interactions.

The examples given here together with the outlook that arises from them underpin the relevance and versatility of the new deposition methodologies based on ESI. Clearly, the chemical control over chemical composition by mass spectrometry methods supports the imaging by SPM greatly, because the complexity of the subject allows for a meaningful interpretation only when the relevant parameters are controlled. As the method further matures, we are convinced that many other high-performance, surface sensitive, analytical methods will make use of the chemical selective, gentle deposition.[16,52,60–64]

## Figures and Tables

**Table 1:** Control of biomolecule conformation in ESIBD.

Parameter	Effect
Gas-phase conformation	Folded – native ESI Unfolded – organic solvent, acidified
Charge state	High – stiff, extended (Fig. 1a, right) Low – soft, compact (unfolded) or native (Fig. 1a, left)
Deposition energy	High – compaction upon landing, fragmentation Low – extended polymer
Surface diffusion	High – self-compaction, 2D folding Low – extended polymer structure
Self-interactions	Polar – self-compaction, 2D folding (Fig. 1b) Unpolar – extended polymer structure
